# Compensatory task-specific hypersensitivity in bilateral planum temporale and right superior temporal gyrus during auditory rhythm and omission processing in Parkinson’s disease

**DOI:** 10.1038/s41598-019-48791-0

**Published:** 2019-09-02

**Authors:** Kjetil Vikene, Geir Olve Skeie, Karsten Specht

**Affiliations:** 1 Department of Biological and Medical Psychology, University of Bergen, Bergen, Norway; 2 Department of Neurology, Haukeland University Hospital, Bergen, Norway; 3 Department of Education, The Arctic University of Norway, Tromsø, Norway; 4 Mohn Medical Imaging and Visualization Centre, Haukeland University Hospital, Bergen, Norway; 5 The Grieg Academy - Department of Music, University of Bergen, Bergen, Norway

**Keywords:** Diagnostic markers, Parkinson's disease

## Abstract

Persons with Parkinson’s disease have general timing deficits and have difficulties in rhythm discrimination tasks. The basal ganglia, a crucial part of Parkinson’s disease pathology, is believed to play an important role in rhythm and beat processing, with a possible modulation of basal ganglia activity by level of rhythmic complexity. As dysfunction in basal ganglia impacts function in other brain areas in Parkinson’s disease during temporal processing, investigating the neuronal basis for rhythm processing is important as it could shed light on the nature of basal ganglia dysfunction and compensatory mechanisms. We constructed an auditory beat-omission fMRI paradigm with two levels of rhythm complexity, to investigate if and where persons with Parkinson’s disease showed abnormal activation during rhythm and omission processing, and whether such activations were modulated by the level of rhythmic complexity. We found no effect of complexity, but found crucial group differences. For the processing of normal rhythm presentations, the Parkinson-group showed higher bilateral planum temporal activity, an area previously associated with the processing of complex patterns. For the omissions, the Parkinson-group showed higher activity in an area in the right superior temporal gyrus previously associated with detection of auditory omissions. We believe this shows a pattern of “hypersensitive” activity, indicative of task-specific, compensatory mechanisms in the processing of temporal auditory information in persons with Parkinson’s disease.

## Introduction

## Rhythm Deficits in Parkinson’s Disease

Persons with Parkinson’s disease (PD) have general timing deficits^1,2^, and perhaps as a particular manifestation of this, they have been found to have difficulties in rhythm discrimination tasks^3–5^. It is unclear if these difficulties are modulated by rhythmic complexity, i.e., if persons with PD have bigger problems with simple, beat-based rhythms^3,4^ or whether it is a more generalized deficit in rhythm discrimination^5^. Basal ganglia, a crucial part of Parkinson’s disease pathology, is believed to play an important role in rhythm and beat processing^6^, and dysfunction in basal ganglia is believed to impact function in widespread cortical areas in PD during temporal tasks, such as sensory and sensory-motor areas^2^. The question of whether timing deficits in Parkinson’s disease is modulated by rhythmic complexity at a neuronal level is therefore important, as it could shed light on the nature of basal ganglia dysfunction and of compensatory mechanisms in Parkinson’s disease^2^.

A difference between simple (isochronous or strongly metric), complex (non-isochronous, weakly metric) and non-metric rhythms is well established in both behavriousl, EEG/ERP and fMRI-studies perception^7–11^, influenced to a large part by the work of Povel & Essens^12,13^. Another rhythm framework posits that hierarchical beat *position* and position *deviations* are central features influencing our perception^14–19^ and speak to how we process rhythmic complexity within a rhythm.

In this study we wanted to investigate differences between persons with Parkinson’s disease (PD) and healthy controls in rhythm perception, and in short, we wanted to investigate if there would be differences between PD and healthy controls in omission elicited activation, across rhythmic context and across position saliency. To this end, we manipulated one simple and one complex rhythm introducing first and second beat position omissions, i.e., removing an auditory onset. In EEG/ERP-studies, an auditory omission potential is a special case of mismatch negativity^20^ not triggered by extrinsic stimuli quality, but rather by the *lack* of stimuli^21^. This means that, at least theoretically, the potentials are purely intrinsic to the subject, i.e. generated by internally generated responses based on expectancy^21^. Mixed M/EEG/fMRI-studies has shown that it is possible to find MMN-like results in fMRI^22–24^. The main source of MMNs and auditory omissions has consistently been located in the intersection of the STG, PT and HG^25–35^, areas crucially involved in rhythm perception.

We made the following predictions: Should contextual complexity modulate neuronal activity differentially in Parkinson’s disease, we would see a bigger between-group difference in activations for omissions in the simple rhythm that for omissions in the complex rhythm. Similarly, we hypothized that should beat position modulate neuronal activity, our second measure of complexity, an omission in the first, most salient, position would again elicit a bigger between-group difference in activations for omissions than for omissions in the second, less salient, beat position. Thirdly, should the rhythm processing differences in Parkinson’s disease be of a more general nature, i.e., independent of level of complexity and saliency, any overall between-group difference would be the same, independent of both contextual complexity and beat-position saliency. We were particularly interested in differences in activation of basal ganliga areas, superior temporal gyrus and planum temporale, as well as in areas in the superior temporal gyrus found to be activated by auditory omissions in previous studies.

## Materials and Methods

### Participants

15 volunteers with PD were recruited with the help of the National Parkinson’s organization of Norway. 15 healthy controls were recruited, matched case wise for age, sex, education level, as well as for musical expertise. In two cases women HCs were matched to male PDs. A minimum Mini Mental Status (MMS)^36^ test score of 24 was set in both groups to exclude patients with cognitive impairment indicative of dementia. All participants reported they were right handed. Unified Parkinson’s disease rating scale III (UPDRS-III)^37^ was done on PDs by medical doctors at the department of neurology at Haukeland University Hospital, and date of diagnosis obtained from the patients themselves. All PD-participants – except one newly diagnosed de novo patient – were in medication regimens (LDOPA, D2-agonists, inhibitors) at the time of the fMRI-scan. Musicians were labelled as such if they had played an instrument on a regular basis for 5 years or more. See Table 1 for an overview of participants. All procedures were approved by the Regional Committee for Medical and Health Research Ethics (REK no 2014/1915) and carried out in accordance with the code of Ethics of the World Medical Association, Declaration of Helsinki. This is the same cohort of participants that has been reported in a previous paper^38^. Before the tests, all participants gave written informed consent to participate in the study. Participants were compensated with 100NOK for participation in this study.

**Table 1:** Group characteristics.

	N (f)	Age (SD/min/max)	Edu (SD/min/max)	MMS (SD/min/max)
**PD**	15 (6/9)	65.6 (12.38/40/81)	14.0 (3.14/9/18)	28.07 (1.16/26/30)
**HC**	15 (8/9)	64.9 (11.33/40/78)	15.2 (1.78/12/18)	28.67 (1.35/25/30)
**Diff**.	t-test	*p* < 0.7	*p* < 0.21	*p* < 0.2
		**UPDRS-III** (SD/min/max)	**Symptoms** (SD/min/max)	**Diagnosis** (SD/min/max)
**PD Group**		17.67 (4.69/11/28)	7.2 (4.32/1/17)	5.47 (3.28/1/13)

PD: Parkinson’s group. HC: Healthy controls. f: Females. mus: Participants with 5+ years playing an instrument. Other columns: Means (standard deviations/minimum/maximum). Edu: Years of education. UPDRS-III: Universal Parkinson’s disease Rating Scale, Part 3. Symptom: Years since symptoms first noticed by participants themselves. Diagnosis: Years since official diagnosis.

### Stimuli construction

Crucial to the questions in this study is question is the concept of complexity in rhythm. EEG/ERP and fRMI-studies on this topic have focused – overtly or covertly – on differential activation of brain areas and networks according to complexity levels of the rhythms precented^7–11^. Studies such as these have focused – overtly or covertly – on differential activation of brain areas and networks due to levels of rhythmic complexity, i.e., simple/strongly metric and complex/weakly metric rhythms, and attentional saliency, i.e., the first onset of a four beat measure is the most attentional salient and represents a primacy effect, while the second, third and fourth beat are weaker, i.e. attentional less demanding, and were, in oddball paradigms, deviations at the first beat position are considered more salient than deviations in the second position

**Figure 1:**
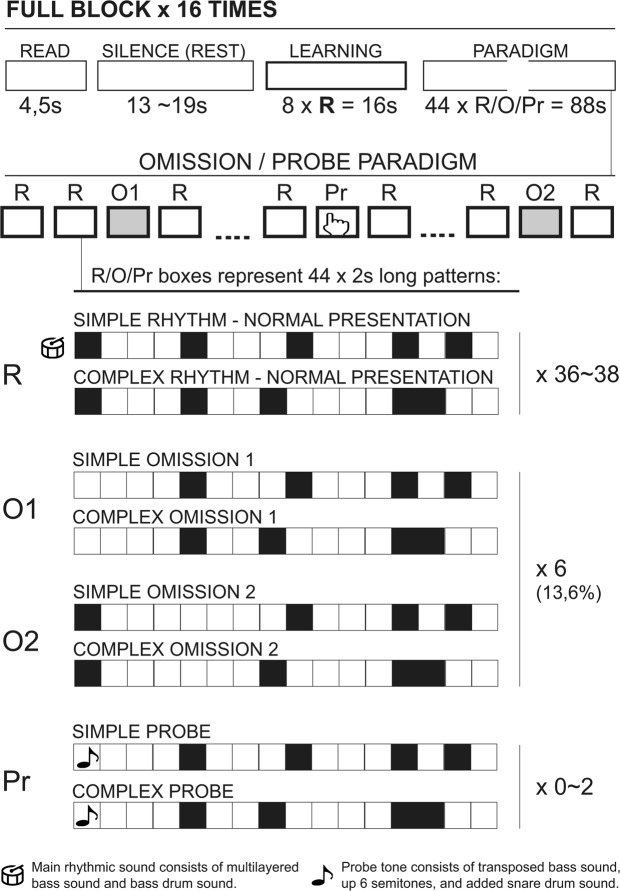
Paradigm/Experimental design. The paradigm was presented in 16 blocks. Top row: The complete block set-up. 4.5 seconds with on-screen instructions (READ) followed by a white screen and silence (REST) followed by a learning period (LEARNING, 8 presentations of the normal variant of the 2 second rhythm patterns) followed by the omission/probe-paradigm (PARADIGM). Second row: The logic of the omission/probe paradigm. R = Normal presentation of the rhythms, simple and complex. O1 and O2 = Omissions at position 1 or 2 respectively, with six occurences in each block. Pr = Probe tone, with zero to two occurences in each block. See text for more details.

In this study, we aimed to combine these two perspectives on rhythm outlined above, and to this end one simple, strongly metric and one complex, weakly metric rhythm were constructed for the experiment (see Fig. 1 for an overview of the paradigm). In both rhythms omissions were introduced either at the first or second beat position. In addition, probe tones – added as an overt attentional task to make sure the participants were focusing on their listening and were awake - were added. The probe tone consisted in a simple shift of the tonality of the bass sound (up 6 half tones) and a change in the snare drum percussive sound characteristic, but without noticeable intensity changes to avoid startle effects. Omissions and probe tones were distributed quasi-randomly across the four versions of each rhythm, with each version containing six omissions. Two versions had three instances of first and first position omissions, while two versions had unequal numbers (four first position/two second position omissions and two first position/four second position omissions respectively).

Probe tones were also distributed unequally across the four versions of each stimuli, with one version containing no probe, two version containing one probe and one version containing two probes. The probe tones were positioned so not to interfere with the omissions, with a minimum of 6 seconds distancing the probe from the previous or following omission. The smallest ISI between omissions was 8,5 seconds, while the longest was 17,5 seconds.

Before each trail, the same written instructions were presented in the goggles (4.5 seconds), followed by blank screen and silences ranging from 13 to 19 seconds, followed by a 16 second long normal presentation of the rhythm (without omissions or probe tones). When the music stimuli began playing, a small cross was presented in the goggles so that the participants had a focus point to minimize head movement. Each of the four versions of the stimuli were presented twice during the scan, with the sequence of stimuli randomized. Total scan time for the task was 33 minutes ((4.5 sec READ + 13~19 sec average SILENCE + 16 s of unmanipulated music + 88 s omission-manipulated stimuli) × 16 stimuli blocks). The final set of 8 different sound files/stimuli was saved as stereo, 16 bit, 44.100 hz .wav-files.

Stimuli was constructed with Steinberg Cubase 7 (http://www.steinberg.net).

### Experimental design

Before scanning, participants underwent training with headphones and stimuli presented from a laptop computer making sure they understood the overt detection task of the probe tones. After training participants where they were placed comfortable in the scanner and fitted with protective in-ear foam plugs, fMRI-compatible headphones with additional physical noise cancellation foamed headphones and fitted with fMRI-compatible video goggles and a response-grip for registering responses pertaining the probe tones. Subjects were given a short reminder of the task, and given three short tests with probe tones to verify that they understood the task, could hear the probe properly and also that button presses were registered. Stimuli were presented using E-Prime (Ver 2.3 Professional, Psychology Software Tools, Pittsburgh, PA).

### Data acquisition, epoching and analysis

fMRI images were acquired using a 3 T scanner (GE Signa Excite 750) with a 32 channel coil. Repetition time (TR) for the EPI-sequence was 1.5 seconds, echo time (TE) was 30 ms, slice thickness 5 mm, with 28 slices interlaced. Pre-processing steps included realignment (0.9 quality, 5 mm smoothing kernel, registered to first image with 2^nd^ degree B-spline), unwarping (using 12 × 12), resliced to mean image, normalized to ICBM-template (with 2 mm^3^ voxel size), smoothing with Gaussian kernel (5 mm^3^ voxels). The 28 slices were temporally aligned to the 13^th^ slice, and high-pass filtering threshold was set at 1/249 Hz cut-off (calculated as the mean between onsets of the 16 main stimuli blocks). Single subject data were analysed by specifying a general linear model, and for the whole scan, movement related variance (realignment parameters) was included in the model as 6 covariates of no interest.

The individual stimuli blocks were divided into epochs and event regressors as follows: First the blocks were divided between the simple and complex rhythm. Each block were epoched as follows: 4,5 seconds of on screen instructions were labeled “READ”. Silences (13–19 seconds random) between each block were *not* epoched and thus served as contrast for all other epochs (“REST”). The first 16 seconds of the rhythmic stimuli were included as a regressor of no interest (labelled “LEARNING”) in the analyses for the current study, but findings related to these have been reported previously^38^. The remaining rhythmic stimuli were epoched in 2-second bins, i.e., the total length of a whole rhythmic pattern. 2 seconds bins containing a probe tone were labelled “PROBE” and blocks where participants made mistakes in grip responses to PROBE-tones were label regressors of no interest and the whole block was excluded from analysis. The rest of each block was split in 2 seconds epochs, labelled “simple_rhythm” and”complex_rhythm” for normal presentation of the beat, “simple_omission_1” and “complex_omission_1” for epochs where the omission was in the first position, and “simple_omission_2” and “complex_omission_2” where the omission was in the second position of the rhythmic pattern.

First level analysis produced contrasts for SIMPLE_RHYTHM, COMPLEX_RHYTHM contrasted to rest, and SIMPLE/COMPLEX/OMISSION/1/2, contrasted to SIMPLE/COMPLEX RHYTHM respectively.

Second-level analysis included a 2 × 2 Anova on the normal presentation of the two rhythms (“simple_rhythm” and “complex_rhythm”), using group as an independent between-group factor, and rhythm as a dependent factor with follow-up t-tests, and a 2 × 2 × 2 Anova on the contrasts of omission > normal presentation, using group as independent between-group factor, and rhythm and position as dependent factors, with follow-up t-tests. Medication type were entered as covariates and interaction covariates into the second level analysis, as were time since diagnosis. FMRI data were preprocessed and analyzed using Statistical Parametric Mapping (SPM12; Wellcome Trust Centre for Imaging, London, UK; http://www.fil.ion.ucl.ac.uk/spm).

## Results

In the 2 × 2 Anova on the normal presentation of the two rhythms (“simple_rhythm” and “complex_rhythm”), we found main effects of listening and group (F(1, 52) = 29,8, family wise error corrected (FWE), p < 0.05, 10voxel cluster-size), while no main effect for rhythm or any interaction effects were found. Follow up t-tests showed an across-groups general effect of listening the normal presentation of the rhythmic bars as activation of bilateral temporal gyri, supplementary motor area, bilateral pre-motor cortex and bilateral inferior occipital lobes (Table 2/Supplementary Fig. 1A).

**Table 2:** General, across-groups effect of normal presentation of the rhythm > REST.

	Region	MNI			Size	*t*
		X	Y	Z		
R	S/MTG	60	−20	−4	3176	14,36
R		48	−10	−10		13,28
R		48	−20	0		12,33
L	S/MTG	−52	−12	−2	2791	12,63
L		−44	−24	2		12,41
L		−54	2	−2		9,89
R	PMC	50	−2	46	213	9,01
L	PMC	−50	−6	52	229	8,81
R	OccInf	38	−82	−12	304	8,66
R		40	−88	−6		7,53
R	SMA	2	0	62	220	7,91
L		−8	−4	68		5,73
L	OccInf	−46	−80	−12	188	7,05
L		−38	−86	−16		6,62
L		−30	−92	−12		6,24
R	PMC	42	−6	58	11	5,58

Between-group t-tests showed that the PD-group showed higher activation of bilateral superior temporal gyri/planum temporale, a small activation of the right hippocampus and higher activation of the left ventromedial prefrontal cortex, relative to the healthy controls (Table 3/Supplementary Fig. 1C).

**Table 3:** Between-group effect of normal presentation of rhythm > REST, PD > HC.

	Region	MNI			Size	*t*
		X	Y	Z		
R	STG/PT	46	−36	18	91	12,35
L	VMPFC	−32	60	−2	100	7,94
L		−26	60	−10		7,39
L		−22	64	−2		5,93
L	STG/PT	−56	−40	22	22	6,32
R	Hippo	38	−6	−20	17	6,03

In the 2 × 2 × 2 Anova on the contrasts of omission > normal presentation, we found main effects for omission and group (F(1, 108) = 25.9, FWE-corrected, p < 0.05, 10voxel cluster-size), while no main effect for rhythm class, position or any interaction effects were found. Follow-up t-tests showed an across-groups general effect of omission in bilateral (but slightly right lateralized) temporal gyri, premotor cortices, supplementary motor areas and right inferior frontal gyrus (Table 4/Supplementary Fig. 1B).

**Table 4:** General, across-groups effect of omissions > normal presentation of the rhythm.

	Region	MNI			Size	*t*
		X	Y	Z		
R	S/MTG	58	−38	8	3091	12,02
R		52	−16	−20		10,05
R		60	−18	−14		9,26
L	S/MTG	−60	−40	10	1475	10,36
L		−46	−40	4		6,96
L		−64	−20	−8		6,83
R	PMC	50	2	48	1861	9,52
R	Operc	40	12	28		7,24
R	IFG	48	24	14		7,05
R	SMA	4	4	62	614	8,67
R		8	16	46		6,74
L		−6	−8	70		6,04
L	PMC	−48	−2	50	231	8,05
R	PFC/MCC	6	34	38	57	6,18
L	MTG	−52	2	−14	45	6,16
L	WM/AntIns	−24	28	−2	54	5,90
R	Precuneus	8	−72	46	49	5,82
R	WM/Occ	30	46	14	81	5,80
L	Calcerine	−8	−96	0	27	5,62
L	Occ	−30	−62	36	36	5,61
L	WM/Occ	−28	−56	26		5,33
L	Cerebellum	−18	−72	−38	42	5,46
R	Thalamus	−12	−4	6	20	5,41
L	PMC	−36	4	30	10	5,18

Between-group t-test showed higher activation of the right superior temporal gyrus in the PD-group relative to the HC-group (Table 5/Supplementary Fig. 1D).

**Table 5:** Between-group effect of Omission > Normal presentation, PD > HC.

	Region	MNI			Size	*t*
		X	Y	Z		
R	STG	62	−24	−2	17	5,99

**Figure 2:**
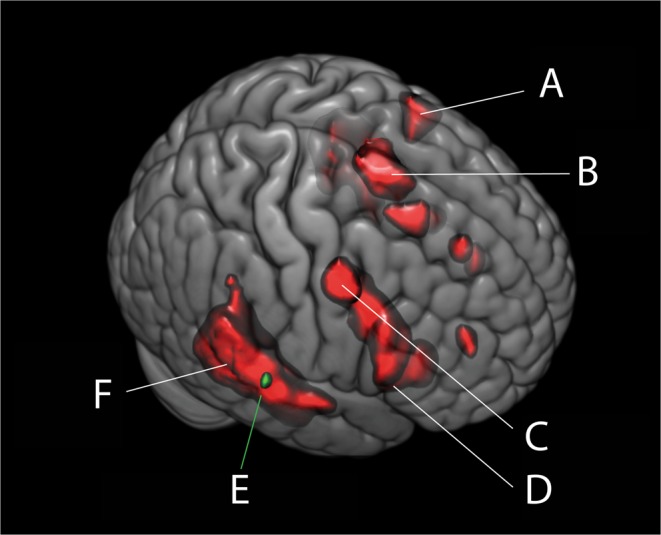
Omission-Activation of the Right Temporal Gyrus, across and between groups. Illustration of activation to omissions in the right temporal gyrus. Red and green areas combined shows across-group activations to omissions, in (**A**) left premotor cortex, (**B**) supplementary motor areas, (**C**) right premotor cortex, extending to (**D**) right inferior frontal gyrus, and in (**F**) bilateral temporal gyrus. Green area alone (**E**) shows the between-group difference, where the Parkinson’s group shows higher activation to omissions than do controls. See Table 2B,D and Supplementary Fig. 1B/2D for complete results. This figure is for illustration purposes only, based on graphical output from the MRIcroGL software.

Abbrivations: STG = Superior Temporal Gyrus, MTG = Middle Temporal Gyrus, PMC = Premotor Cortex, OccInf = Inferior Occipital Gyrus, SMA = Supplementary Motor Area, VMPFC = Ventromedial prefrontal cortex, IFG = Inferior Frontal Gyrus, WM = White Matter, AntIns = Anterior Insula, Occ = Middle Occipital cortex, MFG = Middle Frontal Gyrus, Operc = Operculum, PT = Planum Temporale, Hippo = Hippocampus. See Fig. 2 for an illustration of the results from Tables 2 and 4. See Supplementary Fig. 1 for more detailed results.

Figure 2 shows a masked and combined illustration of the across-group and between-group activations for omission.

Illustration of results from t-tests in Table 4 and Table 5. All results reported as t-tests with family wise error (FWE) correction at p < 0.05, cluster size of 10 voxels. Illustration made with the help of MRIcroGL (http://www.cabiatl.com/mricrogl/).

## Discussion

Our across-group findings for listening to rhythms and omission-related activation are consistent with previous literature on neuronal activation during rhythm processing. Listening to rhythms predictably activated large parts of bilateral temporal gyri, bilateral premotor and supplementary motor areas^7–9^. Omissions activated more posterior parts of the temporal lobes than did the normal presentation of rhythm, and the peak values for omission were very close to those reported in previous studies in healthy populations. In our study, the right peak value in temporal lobes were at MNI 58, −38, 8, comparable to those coordinates reported in two previous studies (MNI 59, −44, 17^35^ and 64, −32, 12^33^ respectively, the first set converted from Tailarach to MNI). Across groups, for the omissions additional activation was seen in right inferior frontal gyrus, consistent with this regions role in attentional control^39^. The overall slight right lateralization in our study is also consistent with previous research on rhythm processing^40^.

These across-group findings make the group-differences interesting, as they potentially say something specific about rhythm processing in the PD-group. In our study, we did not find any interaction between rhythm *complexity* and group neither for the normal presentation of rhythm, the effect of contextual complexity on omission, nor the saliency of the position of the omission. This lack of a significant results does not of course exclude differential activity for simple and complex rhythms between the PD-group and the healthy controls, as reported by previous behavioural studies^3^, and might be due to lack of sensitivity of the fMRI-method for finer grained differences in activation. Although rhythmic complexity, beat-omission and saliency position have previously been used (in ERP) studies to investigate various aspects of rhythm perception^21,41–46^, the findings are inconclusive whether MMN shows difference in saliency (i.e., the influence of beat position) in unattended situations, such as in our study. ERP-MMN studies in PD are scarce (see^47^ for a review), and have predominantly *not* found differences between PD and healthy controls. One study in PD that *did* find differences in (lower) MMN-amplitudes compared to healthy controls^48^ used a fixed inter stimuli interval (ISI) of 1 second – i.e. approximating a steady beat.

For normal presentations of the rhythms, the PD-group showed higher bilateral activation of the planum temporale relative to the control group. The planum temporale plays a role in the analysis and processing of incoming complex sounds at different scales, including musical temporal patterns in the multiple seconds range^49,50^ and is sensitive to patterns and metrical complexity^7,11,40,51–53^. In light of the lack of an effect of rhythmic complexity in our results, an overall higher activation of bilateral planum temporale could nonetheless be a neural expression of a *general* deficit in rhythms processing independent of level of complexity^5^, where hyperactivity of the planum temporale is the reflection of a compensatory mechanism for analysing rhythm in the PD-group. This is still consistent with previous findings of an inverse relationship between the planum temporale and basal ganglia activity in processing rhythms^52^, a relationship that then might be altered in the PD-group due to basal ganglia dysfunction.

One study implicates hippocampus and frontal regions in auditory working memory^54^, and the additional activations in left ventromedial prefrontal cortex and right hippocampus could be indicative of similar compensatory mechanisms, as previous research in healthy subjects indicates increased activity in attentional and working-memory networks with more complex rhythms^51,55^. The activation of these two areas may however be dependent on the attentional task in our study, i.e., detecting the probe tone. An interplay between prefrontal executive control^56^ and hippocampus’ role in shorter term memory processing^57^ has been the focus of recent reviews^58,59^, indicating a dynamic relationship of these two regions in more flexible cognitive operations. As such, activation of these two latter areas could simply be a reflection of more general dysfunction in attention, memory and executive function in PD^60^, underscored by recent research into the connections between the hippocampus and the dopaminergic system in PD^61^.

However, if the PD-group has a generalized problem with rhythm processing^5^, where rhythms are perceived as more complex by the PD-group, increased activation of the planum temporale, the prefrontal cortex while listening to rhythms, and in the right superior temporal gyrus for the omission detection (with a peak value coordinates (62, −24, −2), nearly identical to those found in previous studies^33,35^), this might reflect task-specific (i.e., auditory rhythm specific) altered functional relationships between these areas during rhythm processing in PD.

## Conclusion

The higher bilateral planum temporal activity in the PD group while listening to rhythms and the higher activity in an area in the right superior temporal gyrus previously associated with detection of auditory omissions in healthy subjects, show a pattern of “hypersensitivity” in parts of the auditory system in the PD group previously found to be important for rhythm processing. We take this to be an indication of task-specific, compensatory mechanisms in the processing of temporal auditory information in persons with Parkinson’s disease.

## Supplementary information

Supplementary Figure 1
