# Supplementary material for: Compensatory task-specific hypersensitivity in bilateral planum temporale and right superior temporal gyrus during auditory rhythm and omission processing in Parkinson’s disease
Source: Sci Rep. 2019 Sep 2;9:12623. doi: 10.1038/s41598-019-48791-0 (PMC6718659; doi:10.1038/s41598-019-48791-0)

**Compensatory task-specific hypersensitivity in bilateral planum temporale and right superior temporal gyrus during auditory rhythm and omission processing in Parkinson’s disease.**

**Kjetil Vikene PhD* ^1^, Geir-Olve Skeie PhD ^2^, Karsten Specht PhD ^1,3^**

^1^ Department of Biological and Medical Psychology, University of Bergen, Bergen, Norway

^2^ Department of Neurology, Haukeland University Hospital, Bergen, Norway

^3^ Department of Education, The Arctic University of Norway, Tromsø, Norway

*** Correspondence:**Kjetil Vikene
[kjetil.vikene@uib.no](mailto:kjetil.vikene@uib.no)

**Supplementary Figure 1 title:** General and between-group differences for normal rhythms and beat-omission

**Supplementary Figure 1 legend:** A) General, across-groups effect of normal presentation of the rhythm > REST. B) General, across-groups effect of omissions>normal presentation of the rhythm. C) Between-group effect of normal presentation of rhythm>REST, PD>HC. D) Between-group effect of Omission>Normal presentation, PD > HC. All results reported as t-tests with family wise error (FWE) correction at p<.05, cluster size of 10 voxels.


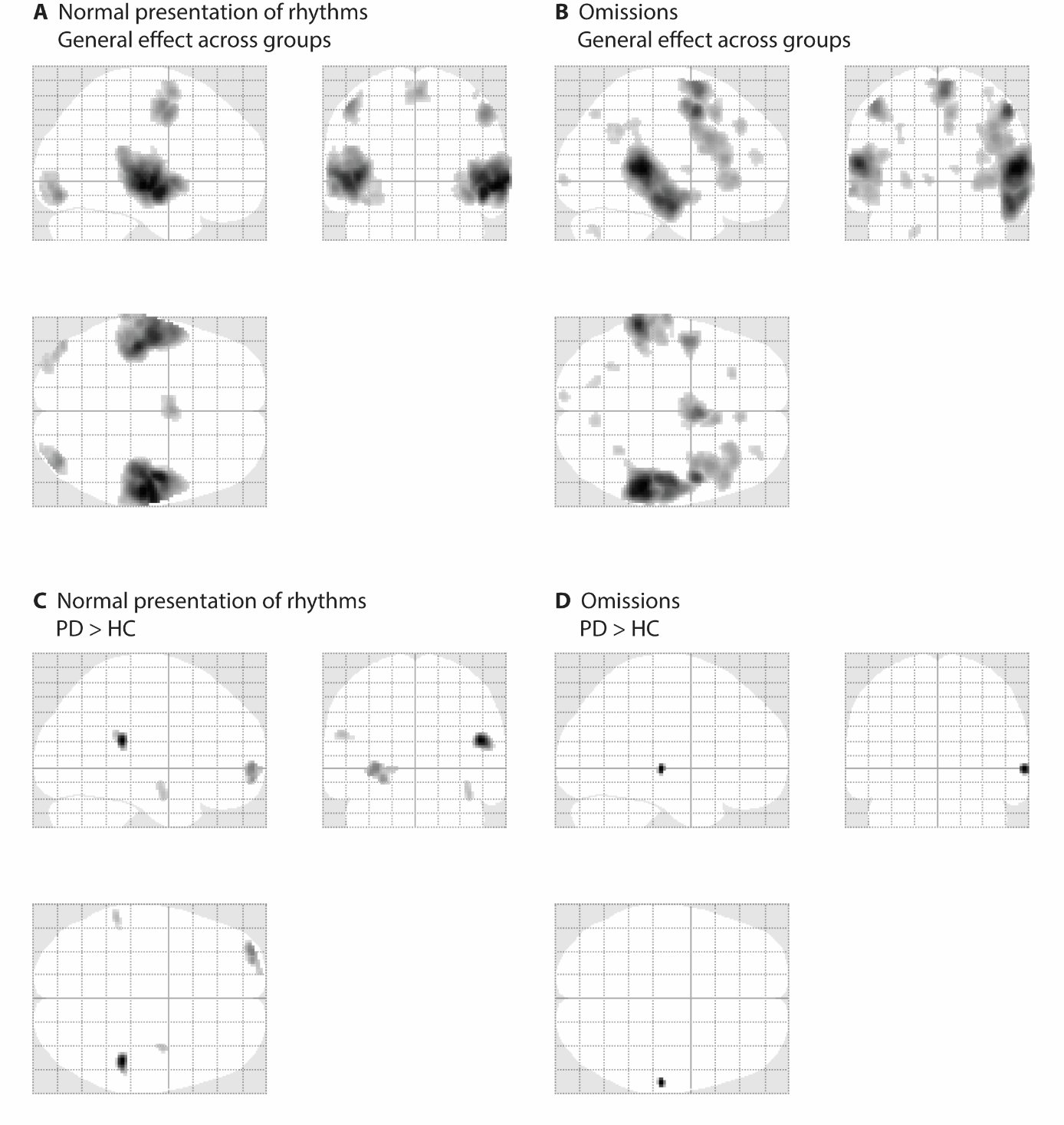

Supplement: Supplementary file 1 — Supplementary Figure 1 [file 41598_2019_48791_MOESM1_ESM.docx]
